# Lack of effects of ooplasm transfer on early development of interspecies somatic cell nuclear transfer bison embryos

**DOI:** 10.1186/s12861-016-0137-6

**Published:** 2016-10-13

**Authors:** L. Antonio González-Grajales, Laura A. Favetta, W. Allan King, Gabriela F. Mastromonaco

**Affiliations:** 1 Department of Biomedical Sciences, University of Guelph, 50 Stone Road E, Guelph, Ontario N1G 2W1 Canada; 2 Reproductive Physiology, Toronto Zoo, 361A Old Finch Avenue, Toronto, Ontario M1B 5K7 Canada

**Keywords:** ATP, Gene expression, Micromanipulation techniques, Mitochondria

## Abstract

**Background:**

Successful development of iSCNT (interspecies somatic cell nuclear transfer) embryos depends on complex interactions between ooplasmic and nuclear components, which can be compromised by genetic divergence. Transfer of ooplasm matching the genetic background of the somatic cell in iSCNT embryos is a valuable tool to study the degree of incompatibilities between nuclear and ooplasmic components. This study investigated the effects of ooplasm transfer (OT) on cattle (*Bos taurus*) and plains bison (*Bison bison bison*) embryos produced by iSCNT and supplemented with or without ooplasm from cattle or plains bison oocytes.

**Results:**

Embryos in all groups were analysed for developmental competence that included cleavage rates, ATP content, and expression of nuclear- and mitochondrial- encoded genes at 8–16 cell stage. Interestingly, no significant differences were observed in embryo development, ATP content, and expression of nuclear respiratory factor 2 (NRF2), mitochondrial transcription factor A (TFAM) and mitochondrial subunit 2 of cytochrome c oxidase (mt-COX2) among groups. Thus, although OT did not result in any detrimental effects on the reconstructed embryos due to invasive manipulation, significant benefits of OT were not observed up to the 8–16 cell stage.

**Conclusions:**

This study showed that a viable technique for OT + SCNT is possible, however, further understanding of the effects of OT on blastocyst development is necessary.

## Background

Ooplasm transfer (OT) has revealed fundamental insights in the field of reproductive biology. Transferring ooplasm to a female gamete allowed the discovery of maturation promoting factor in frog oocytes [1], provided evidence of ooplasmic control on mouse embryonic arrest at the 2-cell stage [2], and helped female patients with a history of embryonic failure [3] and idiopathic infertility [4]. However, OT and other invasive techniques, such as spindle transfer, have been discouraged in human reproduction due to safety and ethical concerns [5, 6]. On the other hand, animal models offer an unique opportunity to investigate the effects of ooplasm-derived components on embryonic development and their interactions with nuclear structures, as in the case of embryos produced by interspecies somatic cell nuclear transfer (iSCNT) [7, 8].

Evidence has shown that incompatibilities between nuclear and mitochondrial genomes in iSCNT embryos have a negative impact on embryo development (reviewed by [9]). It has been proposed that proteins encoded by the nuclear genome are unable to recognize specific sequences of the mitochondrial DNA (mtDNA) due to different taxonomic origin [10]. Furthermore, Zhong et al. [11] suggested that embryonic centrosome dysfunction might be caused by incompatibilities between the ooplasm and the somatic cell. Understanding the interactions between nuclear and ooplasmic compartments is essential to determine the potential causes leading to reduced development or embryonic arrest in iSCNT embryos at early cleavage stages. However, the influence of mitochondrial make-up (heteroplasmy vs xenomitochondrial homoplasmy) on embryo development is complicated with many contradictory findings. iSCNT studies have demonstrated the ability of enucleated oocytes to support first mitotic division with subsequent failure to activate embryonic genes [12, 13]. In wild cattle iSCNT embryos, evidence of increased developmental arrest compared to SCNT controls was observed at the 8–16 cell stage (gaur: [14]; bison: [15]). Therefore, supplementing ooplasm from the same species as the somatic cell into iSCNT embryos could influence embryo development by transferring species-specific proteins, organelles, and other molecules [8, 14, 16]. However, very few reports have implemented OT to investigate its effects on early development of iSNCT embryos.

Sansinena et al. [17] first documented the use of OT in iSCNT embryos and explored its potential applications to overcome the embryonic arrest occurring at early developmental stages observed in a large proportion of iSCNT embryos. Despite the novel approach used in their experiments, one of the main limitations of this technique is the dexterity required to reconstruct and supplement embryos with a small portion of foreign ooplasm. Consequently, implementation of OT with iSCNT might be technically challenging when large numbers of embryos are required. More recently, OT has been carried out in SCNT embryos to determine the effects of the technique on developmental potential and to replenish ooplasm losses as a result of the enucleation procedure [18]. Although no significant differences were found at the blastocyst stage in Gopalakrishna’s study, an improvement in fusion rates and early embryo development was observed in the replenished group.

Previous iSCNT experiments conducted in our laboratory used somatic cell donors from two North American bison subspecies (*Bison bison bison* and *Bison bison athabascae*) and domestic cattle oocytes to investigate potential causes responsible for embryonic arrest commonly observed at the 8–16 cell stage [15, 19]. Numerous analyses performed at this stage of development showed alterations in mitochondrial function (metabolism, apoptosis, transcription) predominantly in bison iSCNT embryos [19]. Recently, we investigated whether incorporation of bison ooplasm into bison iSCNT embryos might affect fundamental processes previously studied, such as energy reserves, gene expression, and development rates. To conduct OT + iSCNT experiments, preliminary results revealed that the additional step required to transfer ooplasm limited the use of OT in conjunction with iSCNT due to increased oocyte lysis and elevated exposure time to environmental factors during micromanipulation. Therefore, the aims of this study were to: 1) establish a 2-step micromanipulation technique to supplement ooplasm derived from cattle oocytes at methaphase II into cattle SCNT embryos, and to 2) determine the effects of cattle and plains bison ooplasm transfer on in vitro embryo development, ATP content, and expression of nuclear and mitochondrial genes in cattle SCNT and plains bison iSCNT embryos.

## Methods

### Experimental design

Four experimental groups were included in this study: 1) cattle SCNT (C), 2) cattle SCNT supplemented with cattle ooplasm (C + OT), 3) plains bison iSCNT (PB), 4) plains bison iSCNT supplemented with plains bison ooplasm (PB + OT). Domestic cattle (*Bos taurus*) oocytes were used as cytoplasts in all groups while plains bison oocytes (*Bison bison bison*) were used only for ooplasm supplementation into iSCNT embryos. Cleavage divisions at 2, 4, and 8–16 cell stages were determined at 36, 50, and 80 h post oocyte activation (hpa), respectively. Each experiment was repeated on 3 biological replicates. Reconstructed embryos at 80 hpa (day 4) showing normal morphology were randomly assigned to either ATP or PCR analyses. A fifth group consisting of domestic cattle embryos produced by in vitro fertilization (C-IVF) techniques using cattle sperm was included only when carrying out qPCR experiments as an additional control. Cleavage rates were estimated based on the total number of reconstructed embryos placed in culture.

### Chemicals

All chemicals were obtained from Sigma-Aldrich Chemical Co. (St Louis, MO, USA) unless otherwise stated.

### Animals

No live animals were used for this study. All animal samples, including ovaries and ear biopsies, were obtained post-mortem from Canadian Food Inspection Agency-approved abattoirs.

### Culture and preparation of somatic cells

Somatic cell donors consisted of ear skin fibroblasts from cattle and plains bison adult males. Post-mortem punch biopsies were collected from local abattoirs. Fibroblasts were cultured as described previously [14]. Somatic cell donors were at passages 3–5 and confluent for 2–3 days.

### Oocyte collections and in vitro maturation (IVM)

Cattle and plains bison ovaries were obtained from local abattoirs. Collection of cumulus-oocytes complexes (COCs) was performed by follicular aspiration into HEPES-buffered Ham’s F-10 supplemented with 2 % steer serum (Cansera International Inc., Rexdale, ON, Canada). Classification of COCs was performed following the inclusion criteria of Leibfried and First [20]. Oocyte in vitro maturation procedures were performed as described previously [19].

### In vitro fertilization (IVF) of cattle oocytes

Frozen-thawed *Bos taurus* semen (EastGen, Ontario, Canada) with known in vitro fertility was used for IVF. Semen was prepared by swim-up in HEPES/Sperm TALP for 45 min at 38.5 °C in a humidified atmosphere of 5 % CO_2_ in air. COCs were washed three times in HEPES/Sperm TALP at 22 h post IVM, and transferred to 80 μl droplets containing fertilization medium (IVF-TALP, Caisson Laboratories, North Logan, UT, USA) supplemented with 20 μg/ml heparin under silicone oil (Paisley Products, Scarborough, ON, Canada). A final concentration of 1 × 10^6^ motile sperm/ml was used to fertilize 25–30 COCs per droplet. Co-incubation with sperm was carried out for 16 h at 38.5 °C in a humidified atmosphere of 5 % CO_2_ in air. Subsequently, groups of 30 presumptive zygotes were placed in 30 μl drops containing modified synthetic oviductal fluid (SOF) medium (Chemicon-Millipore, Billerica, MA, USA) at 38.5 °C in a humidified atmosphere of 5 % CO_2_, 5 %O_2_, and 90 % N_2_ for 4 days as described by Mastromonaco et al. [21].

### Embryo reconstruction and ooplasm transfer by micromanipulation techniques

**Fig. 1:**
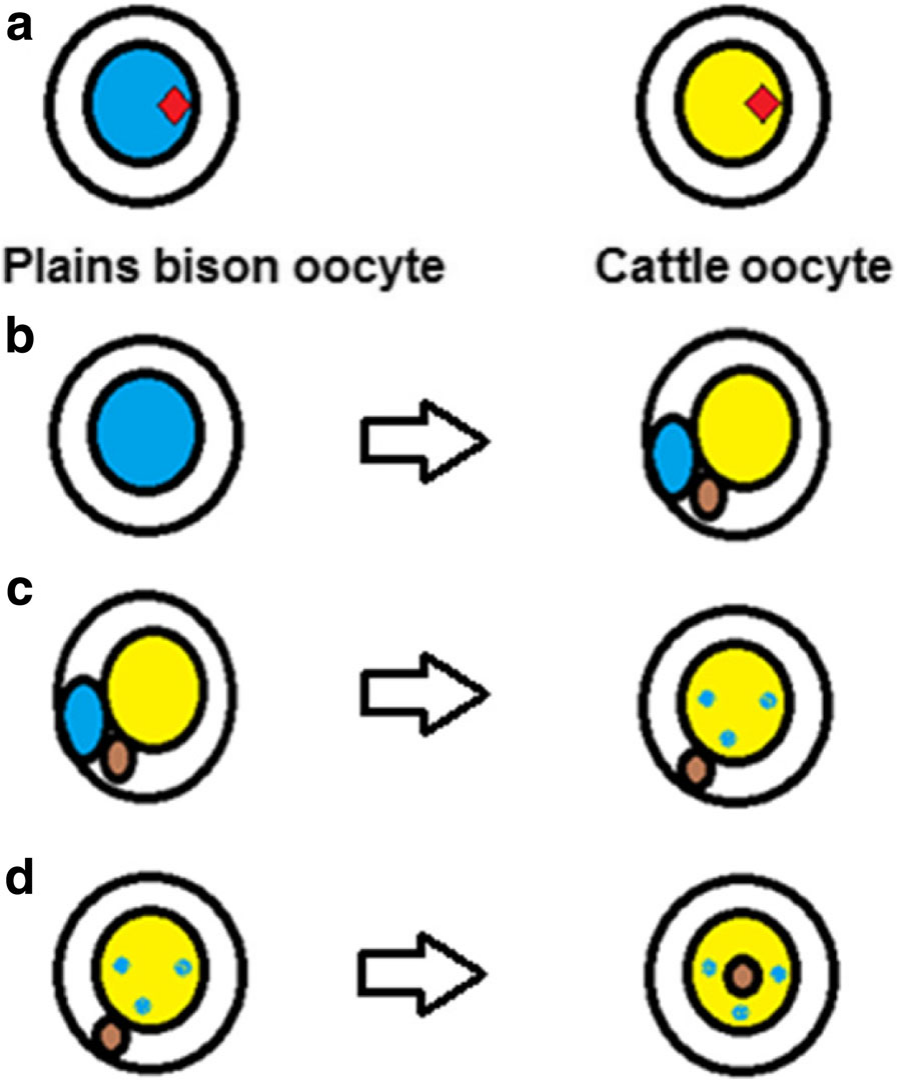
Representation of iSCNT and OT procedures. **a** Large blue and yellow circles represent plains bison and cattle oocytes, respectively. Red diamonds denote a nucleus. **b** A portion of plains bison ooplasm (*small blue circle*) and plains bison somatic cell (*small brown circle*) are introduced in the perivitelline space. **c** Reconstructed embryos + OT after first electropulse (*scattered blue pattern*). **d** Reconstructed embryo after second electro pulse showing successful fusion of the somatic cell

The micromanipulation techniques included three steps: 1) enucleation of recipient oocyte (cytoplast), 2) aspiration of donor somatic cell and small volume of donor ooplasm (ooplast) within transfer pipette, and 3) simultaneous deposition of somatic cell and ooplasm within the perivitelline space. Cattle and plains bison oocytes were selected based on the presence of an extruded polar body and homogenous ooplasm under light microscopy 18 h post-IVM. Enucleation of all denuded oocytes (cytoplasts and ooplasts) and somatic cell transfer were carried out as described by Mastromonaco et al. [21] with some modifications. Cytoplasts and ooplasts were manipulated in 40-μl microdroplets containing HEPES-sperm TALP medium supplemented with 5 μg mL^−1^ cytochalasin B under silicone oil on a stage warmer. Following enucleation, 10 cattle cytoplasts and 2 cattle or bison ooplasts were placed in one 40 μl microdroplet, but at opposite poles to segregate the oocyte types. Donor somatic cells were maintained in a separate 40 μl microdroplet placed 0.5 cm apart. Five somatic cells were aspirated into the end of the transfer pipette (15 μm inner diameter) previously coated with 0.01 % PVA/PVP. Immediately prior to somatic cell transfer, one cell was placed at the pipette tip and 10 to 15 % of either cattle or plains bison ooplasm was aspirated. Deposition of ooplasm and somatic cell within the perivitelline space was performed carefully to avoid lysis of the recipient cytoplast during the procedure. The procedure was repeated until aspiration of more than 70 % of the ooplasm from the ooplast was complete, at which point a new ooplast was selected. Micromanipulations were carried out on an inverted microscope (Leica DM IRB; Leica, Willowdale, ON, Canada). An illustration of a reconstructed embryo including enucleation, transfer of the somatic cell, and ooplasm transfer is shown in Fig. 1.

### Fusion, activation and in vitro culture (IVC) of reconstructed embryos

**Fig. 2:**
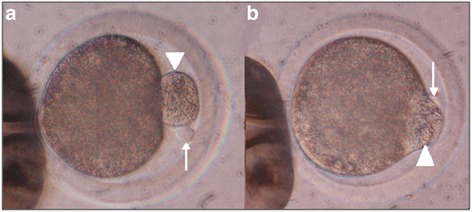
Phase contrast micrographs of oocytes following ooplasm and somatic cell transfer. **a** cattle oocyte + cattle ooplasm and somatic donor cell nucleus, arrow represents the cattle somatic cell while the arrow head denotes the portion of ooplasm being transferred into the perivitelline space; **b** a different oocyte taken 5 min after first DC pulse. Note fusion process between the cattle oocyte and the portion of cattle ooplasm (*arrow head*) with presence of the somatic cell in the back (*thin arrow*)

An Electro Square Porator ECM 830 (BTX Harvard Apparatus, Holliston, MA, USA) was used to fuse the somatic cell and a portion of ooplasm in the reconstructed embryo. Fusion of the reconstructed couplets in the case of C and PB groups employed a single DC pulse at 2.3 kV cm ^−1^ for 23 μs. Each reconstructed embryo from C + OT and PB + OT groups received two pulses applied at a 30 min interval. The first DC pulse was set at 1.5 kV/cm for 20 μs to fuse the transferred ooplasm into the reconstructed embryo. A second DC pulse employing higher voltage and longer exposure time (2.1 kV/cm for 32 μs) was used to fuse the somatic cell into the reconstructed embryo once the portion of transferred ooplasm was not longer visible in the perivitelline space. Fusion in all groups was achieved in a 0.5 mm chamber. The fusion solution contained 0.28 M mannitol, 0.01 % bovine serum albumin (BSA), 100 μM MgCl_2_ and 50 μM CaCl_2_. Micrographs of cattle oocytes before and after fusion of ooplasm are shown in Fig. 2. Oocyte chemical activation protocol was done ~30 min after the second fusion pulse was applied by incubating in HEPES-sperm TALP containing 5 μM ionomycin for 5 min followed by SOF containing 2 M 6-dimethylaminopurine for 4 h at 38.5 °C in 5 % CO_2_ with maximum humidity. Lastly, reconstructed embryos were cultured in 30 μl drops of modified SOF covered by silicone oil for 80 h (hpa) using the same conditions mentioned in the previous section.

### Adenosine triphosphate (ATP) determination

Accumulated ATP was determined by an adenosine 5′-triphosphate quantification assay (Bioluminescent Somatic Cell Assay Kit; Sigma-Aldrich Canada) in individual embryos from all experimental groups. The protocol was performed as described by González-Grajales et al. [19] while solutions were prepared according to the manufacturer’s instructions. Embryos were placed in 10 μl of HEPES-sperm TALP medium, snap frozen and kept at −80 °C until needed. Luminescence reading was performed using a Fluostar Optima plate reader (BMG Labtech, Ortenberg, Germany). Values for ATP content determined in pmol/embryo were generated from a standard curve containing values ranging from 0 to 5 pmoles. A total of 10 embryos per group were included for this analysis except for C + OT which included 13 samples.

### RNA extraction, reverse transcription, and gene expression analysis

Embryos from each experimental group were collected in pools of five at the 8–16-cell stage and washed three times in 0.1 % PBS–PVA, snap-frozen in liquid nitrogen and stored at −80 °C until needed. RNA was isolated from pooled embryos using the miRNeasy Micro Kit (QIAGEN, Inc., Burlington, ON, Canada) following the manufacturer’s instructions with minor modifications, as previously described [19]. RNA samples were reverse transcribed immediately following extraction using the one-step protocol with qScript cDNA Super Mix (Quanta Biosciences, Gaithersburg, MD, USA) following the manufacturer’s instructions. Complementary DNA samples were stored at −20 °C until needed. Quantitative real-time polymerase chain reaction (qPCR) was used to measure mRNA expression profiles of selected genes in each experimental group at the 8–16-cell stage. Each transcript analysis was performed on three biological replicates with three technical replicates for each of them, unless otherwise stated. Two of the chosen transcripts (mitochondrial transcription factor A (TFAM) and nuclear respiratory factor 2 (NRF2) belong to the group of genes related to mitochondrial replication, respiration and DNA transcription, all of which are encoded by the nucleus. The third transcript is encoded by the mitochondria (mt-COX2) and encodes subunit 2 of cytochrome c oxidase (complex IV of the electron-transport chain). Glyceraldehyde-3-phosphate dehydrogenase GAPDH was used as a reference gene for all qPCR experiments. All primers were successfully used in *Bos taurus*. The primer sequences and information are listed in Table 1. Specificity of the primers was checked by sequencing and BLAST analysis, as previously described [19]. Quantitative reverse transcription PCR (RT-qPCR) was carried out using the BIO-RAD CFX96 Real-Time PCR System (BIO-RAD Laboratories, Mississauga, ON, Canada) and products were detected with SsoFastTM EvaGreen Supermix (Bio-Rad Laboratories, Hercules, CA, USA) according to the manufacturer’s protocols, as previously described [19].

**Table 1:** List of primers used for qPCR experiments

Gene	Genbank accession number	Primer sequence	Product size (bp)	Reference
GAPDH	NM_001034034.2	F: 5′-ttcctggtacgacaatgaatt-3′ R: 5′-ggagatggggcaggactc-3′	131	[15]
mt-COX2	3283880	F: 5′-attctgcccgccatcatc-3′ R: 5′-cgtagctcccctggcttt-3′	203	[19]
TFAM	NM_001034016.2	F: 5′-ccgaaaagacctcgctca-3′ R: 5′-tctcgtccaacttccatcatt-3′	221	[19]
NRF2	AB162435	F: 5′-tccaacctttgtcgtcatca-3′ R: 5′-ttgcccgtagctcatctctt-3′	174	[14]

### Statistical analysis

Fusion and development rates at 2, 4, 8–16 cell stages were compared using Chi-square in all experimental groups. ATP quantification and gene expression levels were analysed using One-way ANOVA. A probability of <0.05 was considered to be significant. Data were analyzed through the computer program RSTUDIO Version 0.97.551/IC 10.1 (Rstudio, Boston, MA, USA). Data are presented as mean ± SEM.

## Results

### Fusion rates

The percentage of oocytes with fused ooplasm within 30 min in C + OT and PB + OT groups was 93.0 ± 5.1 and 92.4 ± 2.0, respectively. After the second pulse (30 min waiting period), rates for fusion of the somatic cell in the former group was 40.76 ± 4.0 (*n* = 117), while for the latter was 53. 9 ± 0.7 (*n* = 75). Fusion rates for C and PB were 42.5 ± 3.9 (*n* = 91) and 41.1 ± 3.0 (*n* = 111), respectively. There was a significant difference in the proportion of fused oocytes between PB and PB + OT (*p* = 0.01), however no significant differences were found when C and C + OT groups were compared (*p* = 0.6).

### In vitro development of reconstructed embryos

The first three cleavage divisions in all groups (C, C + OT, PB, PB + OT) were monitored to identify differences in development. No significant differences in the proportion of embryos among groups at 2, 4, and 8–16 cell stages were found (*p* > 0.05). Developmental rates for 2, 4, and 8–16 cell stage ranged between 87.5- 91.7 (*p* = 0.81), 76.0–83.5 (*p* = 0.76), and 57.6–61.2 % (*p* = 0.96), respectively (Table 2).

**Table 2:** Percentage of reconstructed embryos developed to the 2, 4, and 8–16 cell stage

Stage of development	C [*n* = 85]	C+ OT [*n* = 80]	PB [*n* = 85]	PB + OT [*n* = 50]	*P*-value
2-cell stage^a^	91.7 ± 4.0	87.5 ± 2.4	88.2 ± 3.5	88.0 ± 2.4	0.81
4-cell stage^a^	83.5 ± 2.5	80.0 ± 4.9	80.0 ± 6.8	76.0 ± 4.9	0.76
8–16 cell stage^a^	57.6 ± 4.7	61.2 ± 8.1	60.0 ± 7.3	58.0 ± 7.4	0.96

^a^values are reported as mean ± SEM

### ATP quantification

**Fig. 3:**
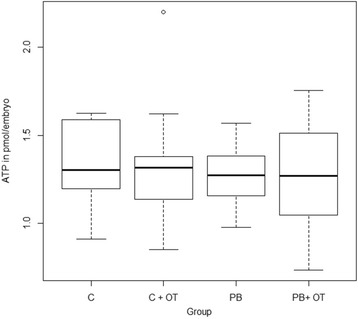
ATP quantification in reconstructed embryos at 8–16 cell stage with or without OT

Quantification of ATP was performed in single embryos at the 8–16 cell stage to determine changes in ATP accumulation as a result of ooplasm transfer. ATP content (pmol/embryo) did not differ among groups (*p* > 0.05). ATP values in C, C + OT, PB, PB + OT were 1.35 ± 0.23, 1.31 ± 0.34, 1.27 ± 0.18, and 1.26 ± 0.31 pmol/embryo, respectively. ATP accumulation from all groups is shown in Fig. 3.

### Expression profiles of nuclear and mitochondria-encoded genes

**Fig. 4:**
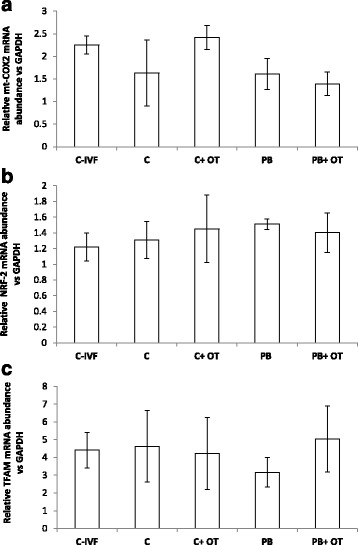
Gene expression profiles of mt-COX-2 (**a**), NRF-2 (**b**), and TFAM (**c**) in IVF and reconstructed embryos at the 8–16 cell stage

Analysis of expression profiles of gene transcripts was determined by qPCR. Expression of mRNA in embryos supplemented with or without ooplasm and IVF embryos at the 8–16 cell stage was compared to the house-keeping gene GAPDH. The expression profiles for mt-COX2 showed no significant difference among groups (*p* = 0.36). In the case of the other two genes similar expression levels were found for NRF2 (*p* = 0.93), and TFAM (*p* = 0.80) in all groups. Expression profiles for all genes are shown in Fig. 4.

## Discussion

It has been hypothesized that ooplasm containing components matching the same taxonomic origin as the somatic cell in iSCNT embryos could influence nuclear-ooplasmic interactions and developmental outcomes [14]. However, limited information is available regarding the effects of OT on early embryo development of iSCNT embryos. Sansinena et al. [17] showed that supplementation of caprine ooplasm in iSCNT embryos (domestic cattle cytoplast x caprine somatic cell) yielded low fusion and cleavage rates, and poor blastocyst rates (0 %). Their results might have been attributed to technical difficulties since the study lacked a control group (goat ooplasm transferred into goat SCNT embryos). On the other hand, studies applying OT to in vitro produced human and animal embryos have revealed contradictory results since both detrimental and advantageous effects have been attributed to OT [3, 22]. Our investigation showed that a portion of ooplasm transferred into SCNT and iSCNT embryos had no effects on embryo development rates, ATP content, or expression of mitochondrial and nuclear genes in embryos up to the 8–16 cell stage.

Previous studies have successfully transferred and fused ooplasm into oocytes recruited for parthenogenesis and iSCNT experiments [17, 23]. However, combining OT and SCNT requires excessive micromanipulation that may lead to oocyte damage. The technique applied in our experiments reduced the micromanipulation steps resulting in acceptable reconstruction rates evidenced by maintenance of normal ooplasm architecture and high ooplasm fusion rates. Low fusion rates of the somatic cell, both in control and treatment groups resulted in loss of 50 % of reconstructed embryos. Interestingly, more couplets were fused in the PB + OT group than PB group which might be related to increased proximity between the plains bison somatic cell and the oolemma caused by an increase in total ooplasmic volume. Conversely, this was not evident in the C + OT group. Experiments conducted to investigate the causes of low fusion rates obtained in this study did not yield any improvement in fusion rates; therefore, it remains unclear if higher ooplasm volumes resulting from OT enhances fusion in embryos reconstructed by SCNT.

Animal and human OT studies have recommended the transfer of approximately 10 to 15 % of ooplasm volume [3, 23]. A similar approach was used in this study to assess the effects of OT on development, ATP content, and gene expression of iSCNT embryos. Optimal ATP content in oocytes and embryos in different species has been associated with embryo competence since this molecule is involved in several cellular processes during the preimplantation stages (reviewed by [24]). It has been hypothesized that transfer of ooplasm or somatic mitochondrial extract could enhance energy levels in the newly formed embryo resulting in improved development [25, 26]. A study conducted by Van Blerkom et al. [27] showed that fertilized mouse oocytes microinjected with somatic mitochondria accumulated 30 % more ATP 36 h post injection with gradual increases throughout development. The results from our study showed no differences in ATP content among groups 4 days after ooplasm supplementation. This may be explained by the ability of the bovine oocyte to target and eliminate foreign mitochondria at the 8–16 cell stage [28]. Furthermore, the ATP findings obtained in this experiment correlate with embryo development rates at the 2, 4 and 8–16 cell stages (*p* > 0.05).

Improvement in developmental outcomes by OT has been linked to abundance of mRNAs, functional mitochondria, and presence of essential molecules [29]. Mitochondrial transfer into oocytes and embryos has been used to determine the effects of mitochondrial heteroplasmy on subsequent development [27]. Evidence has suggested that addition of mitochondria sharing the same taxonomic origin as the somatic cell might lead to disturbances in iSCNT embryos. This concept was illustrated by Hua et al. [30] who reported impaired development in ovine iSCNT embryos (i.e., ovine somatic cell and domestic cattle cytoplast) microinjected with mitochondria isolated from ovine granulosa cells. Significantly fewer embryos were able to progress through the 8-cell stage in comparison with iSCNT embryos injected with mitochondria derived from domestic cattle granulosa cells. Their findings demonstrated that heteroplasmy (close to 0.29 %) contributed to poor development in ovine iSCNT embryos injected with ovine somatic mitochondria. Furthermore, the same authors reported downregulation of pluripotency genes, such as SOX-2 and OCT4, in conjunction with upregulation of TFAM and POLRMT, which are genes involved in mitochondrial replication and transcription [31, 32].

In the last decade, revolutionary disciplines, such as transcriptomics have allowed comprehension of complex processes occurring during early development [33]. Quantification of transcripts in embryos has been used to assess embryo developmental competence in vitro and in vivo [34, 35]. However, very few OT reports have included PCR studies to understand the interactions taking place between ooplasmic and nuclear components. Thus, we studied expression profiles of genes involved in mitochondrial events such as respiration, transcription and replication (e.g. mt-COX2, TFAM, and NRF2). Our results showed similar gene expression profiles among groups. Moreover cattle IVF embryos were included in this study as an additional control group to investigate expression of these genes at the 8–16 cell stage. Findings from the C-IVF group were in accordance with previous investigations, which documented low expression levels of mitochondrial replication and transcription factors [36, 37]. Interestingly, our study revealed that plains bison ooplasm transferred into bison iSCNT embryos did not alter gene expression of mitochondrial and nuclear encoded genes. Our results suggest that mitochondrial events depending on nuclear and ooplasmic communication are not affected, either positively or negatively, by supplementation of either cattle or plains bison ooplasm at the 8–16 cell stage. In addition, accessibility to plain bison oocytes was limited and restricted to several weeks. Therefore, the number of samples employed in these experiments could also be a limiting factor in establishing significant differences among groups. Further experiments are required to address thesequestions and determine if the embryo arrest in bison iSCNT embryos could be ameliorated by implementation of OT.

## Conclusions

Application of OT in iSCNT experiments can provide valuable information to understand complex interactions occurring between nuclear and ooplasmic components in reconstructed embryos. However, confounding factors in the ooplasm may mask the beneficial or deleterious effects of supplementing mitochondria contained in a portion of ooplasm. Although our study found no differences at the 8–16 cell stage, further studies are required to understand whether the potential effects associated with OT can be observed at later cleavage stages. Microinjection of mitochondria extract isolated from bison somatic cells, easily available by cell culture, and mature oocytes might provide additional information regarding the role of this organelle in nuclear-ooplasmic incompatibilities.

## Abbreviations

ATP: Adenosine triphosphate
BSA: Bovine serum albumin
C: Cattle embryos produced by somatic cell nuclear transfer
C + IVF: Cattle embryos produced by in vitro fertilization
C + OT: Cattle embryos produced by somatic cell nuclear transfer supplemented with cattle ooplasm
COCs: Cumulus-oocytes complexes
GAPDH: Glyceraldehyde 3-phosphate dehydrogenase
hpa: Hours post oocyte activation
iSCNT: Interspecies somatic cell nuclear transfer
IVC: in vitro culture
IVF: in vitro fertilization
IVM: in vitro maturation
mt-COX2: mitochondrial subunit 2 of cytochrome c oxidase
mtDNA: mitochondrial DNA
NRF2: Nuclear respiratory factor 2
OT: Ooplasm transfer
PB: Plains bison embryos produced by interspecies somatic cell nuclear transfer
PB + OT: Plains bison embryos produced by interspecies somatic cell nuclear transfer supplemented with bison ooplasm
PBS: Phosphate buffered saline
PCR: Polymerase chain reaction
PVA: Polyvinyl alcohol
PVP: Polyvinylpyrrolidone
SCNT: Somatic cell nuclear transfer
SOF: Synthetic oviductal fluid
TALP: Tyrode’s albumin lactate pyruvate
TFAM: Mitochondrial transcription factor A
